# Lower extremity peripheral nerve block for total knee arthroplasty in a patient with chronic inflammatory demyelinating polyneuropathy: A case report

**DOI:** 10.1097/MD.0000000000037601

**Published:** 2024-03-29

**Authors:** Aka (Nakamura) Inaba, Yoshiaki Ishida, Yoshie Toba

**Affiliations:** a Department of Anesthesiology, Seirei Hamamatsu General Hospital, Hamamatsu, Japan; b Department of General Internal Medicine, Seirei Hamamatsu General Hospital, Hamamatsu, Japan; c Department of Critical Care Medicine, Tokyo Metropolitan Bokutoh Hospital, Sumida-ku, Tokyo, Japan.

**Keywords:** chronic inflammatory demyelinating polyneuropathy, muscle relaxants, peripheral nerve block, postoperative pain, regional anesthesia

## Abstract

**Rationale:**

Chronic inflammatory demyelinating polyneuropathy (CIDP) is an autoimmune disorder that affects the peripheral nerves, leading to weakness and sensory symptoms. CIDP is a rare disease, and few studies have reported on anesthetic management in patients with this condition, especially the peripheral nerve block (PNB). Therefore, a safe and standardized anesthetic approach remains to be established. This case report aims to address this gap in evidence by documenting our experience with PNB in a patient with CIDP undergoing surgery. It contributes significantly to expanding the range of anesthetic options and enhancing perioperative outcomes for patients with CIDP.

**Patient concerns:**

A 58-year-old woman diagnosed with CIDP was scheduled to undergo left total knee arthroplasty due to osteonecrosis. We anticipated postoperative pain and aggravation of neurological symptoms.

**Diagnosis:**

CIDP.

**Interventions:**

To manage the postoperative pain, we planned a combination of general anesthesia (GA) and lower extremity PNBs, viz. the tibial and femoral nerve blocks, supplemented with intravenous patient-controlled analgesia. An initial dose of fentanyl 50 µg was administered as analgesia. The tibial and femoral nerves were identified using a peripheral nerve stimulator in conjunction with an ultrasonic echo device while the patient was conscious, to minimize the risk of nerve injury. The tibial and femoral nerve blocks were performed with 20 mL of ropivacaine (0.25%) and dexamethasone 2.2 mg, respectively. Subsequently, we administered fentanyl and ketamine and initiated continuous infusion of remifentanil. Thereafter, propofol 120 mg was administered intravenously over a span of 1 minute, followed by continuous infusion at 4 mg/kg/h. Upon confirming loss of consciousness, we induced GA using a supraglottic airway device without using muscle relaxants. For postoperative analgesia, we administered acetaminophen 1000 mg.

**Outcomes:**

The patient experienced no pain immediately after surgery, and good analgesia was achieved subsequently without worsening of sensory symptoms during rehabilitation.

**Lessons:**

We achieved effective anesthetic management in a patient with CIDP by combining GA with nerve stimulation and ultrasound-guided PNB. It is crucial to devise a personalized anesthesia plan that focuses on the patients’ safety and comfort while minimizing risk in patients with CIDP.

## 1. Introduction

Chronic inflammatory demyelinating polyneuropathy (CIDP) is an autoimmune disorder that affects the peripheral nerves, leading to weakness and sensory symptoms.^[1]^ Since CIDP is a rare disease, few studies have reported on anesthetic management in patients with this condition, especially the peripheral nerve block (PNB).^[2,3]^ Therefore, a safe and standardized anesthetic approach remains to be established. In this context, we present the anesthetic management plan for total knee arthroplasty (TKA) in a patient with CIDP that entailed the combination of general anesthesia (GA) with nerve stimulation and ultrasound-guided PNB, viz. the tibial nerve block (TNB) and femoral nerve block (FNB). The patient provided written consent for the publication of this case report.

## 2. Case presentation

A 58-year-old female patient (height: 161 cm, weight: 64.9 kg) with a medical history of CIDP was undergoing treatment with intravenous immunoglobulin for neuropathy, which manifested predominantly as sensory disturbance in both lower limbs. She was scheduled to undergo left TKA due to osteonecrosis in the left medial femoral condyle. We opted for a combination of GA and lower extremity PNB with the TNB and FNB, supplemented with intravenous patient-controlled analgesia, since we anticipated postoperative pain and aggravation of neurological symptoms.

We performed noninvasive blood pressure and continuous skin temperature monitoring, in addition to electrocardiography and pulse oximetry throughout the procedure. An initial dose of fentanyl 50 µg was administered for analgesia. We employed a linear ultrasound probe (HFL50, SonoSite, Bothell, WA) and a 5-cm, 22-G needle (Stimuplex Ultra.360, B-Braun, Tochigi, Japan) to administer the PNBs while the patient was conscious, to minimize the risk of nerve injury. The tibial and femoral nerves were identified using a peripheral nerve stimulator and an ultrasonic echo device. The TNB and FNB were performed with 20 mL of ropivacaine (0.25%) and dexamethasone 2.2 mg, respectively. Subsequently, we administered fentanyl 50 μg and ketamine 20 mg and initiated continuous infusion of remifentanil at 0.26 μg/kg/min. Thereafter, propofol 120 mg was administered intravenously over a span of 1 minute, followed by continuous infusion at 4 mg/kg/h. Upon confirming loss of consciousness, we induced GA using a supraglottic airway device without the use of muscle relaxants. Acetaminophen 1000 mg was administered for postoperative analgesia at the end of the surgery. Additional fentanyl and ketamine were not administered, and all vital signs were stable during surgery. The duration of surgery was 90 minutes and that of anesthesia was 121 minutes.

After extubation, the patient was transferred to the post-anesthesia care unit. In the post-anesthesia care unit, the patient indicated a pain score of 0 on the numeric rating scale. No deterioration in sensory symptoms was observed in any extremity, except for the lower left limb, which was affected by the PNBs. In the general ward, the numeric rating scale score remained consistently below 3 using intravenous patient-controlled analgesia comprising fentanyl 1600 μg (32 mL), droperidol 5 mg (2 mL), and saline 66 mL, along with acetaminophen 1000 mg. Sensory symptoms were not exacerbated during postoperative rehabilitation but remained similar to their preoperative state. The patient was discharged on the 18^th^ postoperative day. Both lower limbs exhibited muscle weakness on the 64^th^ postoperative day. A neurologist determined that the PNBs were probably not the direct cause of the CIDP exacerbation, but that it was triggered by a perioperative stress reaction, owing to the difference in the timing of the exacerbation event.

## 3. Discussion

Postoperative analgesia for TKA is critical for early mobilization and rehabilitation.^[4]^ Typically, the anesthetic approach for TKA combines GA with regional anesthesia for postoperative pain management. However, the rarity of CIDP has precluded the establishment of guidelines for anesthetic management in these patients. The attendant anesthetic challenges in patients with CIDP include neuromuscular weakness, autonomic neuropathy, residual paralysis following muscle relaxant use, delayed recovery from neuraxial blockade, and possibility of prolonged mechanical ventilation.^[5]^ Hence, we prioritized regional anesthesia with PNB to minimize the risk of nerve injury and opted for GA without muscle relaxants. During the PNB procedure, we closely monitored the nerve itself and neurological symptoms using a peripheral nerve stimulator and an ultrasonic echo device. Immediately following surgery, the patient reported no pain and was comfortable with postoperative life and rehabilitation, demonstrating the effectiveness of the PNB. Notably, there was no exacerbation of the patient underlying sensory symptoms, affirming the safety of our anesthetic strategy in the context of CIDP. These positive postoperative outcomes demonstrate the potential of a personalized anesthesia plan while minimizing risk in patients with complex conditions such as CIDP.

The safety of regional anesthesia for patients with CIDP remains uncertain, since the existing evidence has been derived solely from isolated case reports. Some studies consider neuraxial anesthesia, including spinal and epidural anesthesia, to be safe in patients with CIDP, reporting no significant complications.^[5–9]^ Conversely, there are also reports of patients with CIDP experiencing sustained or worsened neurological symptoms shortly after undergoing surgery.^[2,10]^ The hypothesis of “double crush phenomenon” states that a single axon that is compressed in one area becomes susceptible to damage in another area, resulting in impaired nerve function.^[11]^ Theoretically, the double crush phenomenon may increase these patients’ susceptibility to post-neuraxial anesthesia nerve injury.^[2]^ Furthermore, inadequate postoperative pain management could amplify symptoms during the perioperative phase. Wells et al^[2]^ chose combined spinal-epidural anesthesia (CSEA) for ankle surgery. However, as the effect of CSEA diminished, the patient requested lower extremity PNBs due to the increase in the pain score. On the fourth postoperative day, the patient noted deterioration of her baseline CIDP symptoms, which persisted for 4 months. However, the cause of the worsening symptoms, that is, CSEA or PNB, was unclear. Persistent pain after PNBs could have also acted as a potential aggravating factor.^[12]^

We combined GA with lower extremity PNBs for pain control since we anticipated intense postoperative pain after TKA. Recent studies suggest that the adductor canal block and infiltration between the popliteal artery and capsule of the knee (IPACK) offer safe and effective pain management following TKA.^[13,14]^ However, the safety of these PNBs, which involve drug administration without direct peripheral nerve visualization, is not assured in patients with CIDP. Notably, one study documented delayed foot drop due to the spread of the local anesthetic agent in adductor canal block and IPACK.^[15]^ In addition to the above-mentioned study,^[2]^ one study documented no exacerbation of CIDP symptoms after femoral and ischial nerve blocks for ankle surgery.^[3]^ In our case, the ultrasonic echo device and peripheral nerve stimulator facilitated accurate tibial and femoral nerve identification and appropriate determination of the drug administration distance, minimizing the risk of nerve injury. We also adopted an extra cautious approach to prevent neurological damage. We selected ropivacaine due to its reportedly lower neurotoxicity compared to other local anesthetics^[16,17]^ and used it in combination with dexamethasone to extend the local anesthetic effect for postoperative analgesia, avoiding high concentration ropivacaine.

Neuromuscular disorders are variable and can be classified into groups, such as the M (myopathy and muscular dystrophy), MM (mitochondrial or metabolic myopathy), and N (neurodegenerative, peripheral neuropathy, or spinal muscular atrophy disorder).^[10]^ CIDP is classified as a peripheral neuropathy. Patients with certain neuromuscular disorders exhibit heightened sensitivity to muscle relaxants.^[18]^ Studies have reported that the muscle relaxant effect was prolonged in CIDP.^[19,20]^ Although sugammadex could potentially reverse the muscle relaxant effect safely in patients with CIDP,^[21]^ there is no definitive consensus on its sensitivity to muscle relaxants in these patients. Hence, we refrained from using muscle relaxants in the present case.

It is important to acknowledge the limitations of this case report to ensure a comprehensive understanding of our findings. First, the outcomes presented are based on a single-patient experience, which may limit the generalizability of our results to all patients with CIDP. The complexity and variability of CIDP symptoms among individuals necessitate caution when extrapolating these results to the broader CIDP population. Second, while our anesthetic approach, which combined GA with PNB, was effective for our patient, the absence of a control group or comparative analysis with other anesthetic techniques prevents us from conclusively establishing the superiority or equivalence of this method. Lastly, the specific anesthetic agents and techniques were chosen based on the patient condition and the surgical team expertise, which might not be readily available or preferred in all clinical settings. This factor could affect the replicability of our approach in different medical centers or regions.

## 4. Conclusions

In summary, we achieved effective anesthetic management of a patient with CIDP undergoing TKA by combining GA with nerve stimulation and ultrasound-guided PNB. CIDP is rare and few guidelines cover its anesthetic management. Therefore, it is crucial to devise a personalized anesthesia plan that focuses on the patients’ safety and comfort, in addition to performing close monitoring and judicious use of medications.

## Acknowledgments

We would like to thank Editage (www.editage.com) for the English language editing of this manuscript.

## Author contributions

**Conceptualization:** Yoshiaki Ishida.

**Data curation:** Aka (Nakamura) Inaba, Yoshiaki Ishida.

**Supervision:** Yoshie Toba.

**Writing – original draft:** Aka (Nakamura) Inaba, Yoshiaki Ishida.

**Writing – review & editing:** Aka (Nakamura) Inaba, Yoshiaki Ishida, Yoshie Toba.
